# Clinical Comparison of OC-Sensor Pledia and Phadia 250 for Fecal Calprotectin Testing

**DOI:** 10.3390/diagnostics14222490

**Published:** 2024-11-07

**Authors:** Eunju Shin, Jong Do Seo, Hee Sook Shim, Hanah Kim, Mina Hur, Yeo-Min Yun, Hee-Won Moon

**Affiliations:** Department of Laboratory Medicine, Konkuk University School of Medicine, Seoul 05030, Republic of Korea

**Keywords:** OC-Sensor, calprotectin, Phadia 250, agreement, performance, inflammatory bowel disease

## Abstract

**Background:** The fecal calprotectin (f-Cal) test is a convenient method used for differentiating inflammatory bowel disease (IBD) from functional bowel disorders. The OC-Sensor Pledia (OC-FCa; Eiken Chemical Co., Tokyo, Japan) is a latex agglutination turbidimetric immunoassay used for f-Cal measurements. We evaluated the clinical performance of OC-FCa and compared the f-Cal levels between OC-FCa and Phadia 250 (Thermo Fisher Scientific, Freiburg, Germany). **Methods:** We collected 278 stool samples; of these, 158 were taken from patients with suspected IBD, and 120 were taken from healthy individuals. We analyzed the f-Cal distribution in each group and compared the clinical performance and agreement between OC-FCa and Phadia 250. **Results:** The f-Cal of patients with IBD was significantly different from that of patients without IBD for both OC-FCa and Phadia 250 (*p* < 0.0001 and *p* < 0.001, respectively). The concordance between OC-FCa and Phadia 250 was 82.3%, with moderate agreement (kappa = 0.644, 95% confidence interval = 0.525–0.763). OC-FCa and Phadia 250 showed a high correlation (r = 0.90); their diagnostic performance showed moderate accuracy (AUC = 0.873 and 0.866, respectively) and had no significant difference (*p* = 0.616). **Conclusions:** Both OC-FCa and Phadia 250 showed a high correlation and good clinical performance. F-Cal measured using OC-FCa was reliable for initial differentiation between patients with IBD and without IBD. Therefore, OC-FCa and Phadia 250 could be alternative devices for measuring f-Cal depending on the laboratory situation.

## 1. Introduction

The incidence and prevalence of inflammatory bowel disease (IBD) vary depending on the ethnicity, region, food, and environment [1]. However, the number of patients with IBD is increasing, especially in industrialized countries [1,2]. Since abdominal pain is one of the most common symptoms of IBD, distinguishing between IBD and functional bowel disorders is challenging in clinical practice [3]. Although endoscopy and biopsy are the gold standards for IBD diagnosis [2,3,4], these procedures are invasive for patients and complicated for doctors.

Calprotectin is a major cytosolic protein in neutrophils and is present in different concentrations in serum, feces, and other body fluids, depending on the level of inflammation [2,3,4,5]. Moreover, fecal calprotectin (f-Cal) is well known for its close correlation with IBD [3,4,5,6]. Measuring f-Cal is a simple test for differentiating IBD from functional bowel disorders because of its low cost, non-invasiveness, stability at room temperature, and homogenous distribution within feces [5,6].

The OC-Sensor Pledia (OC-FCa; Eiken Chemical Co., Tokyo, Japan) is a widely used fecal immunochemical test (FIT) for screening fecal hemoglobin (f-Hb) [7,8]. Eiken Chemical Co., Ltd., recently developed a latex agglutination turbidimetric immunoassay device for f-Cal. It uses the same analyzer and collection device for f-Hb and f-Cal [9,10]. Therefore, OC-FCa can increase laboratory efficiency without double sample collection [10]. However, only two studies have evaluated f-Cal using OC-FCa [10,11].

We analyzed the distribution of f-Cal concentrations in healthy populations and patients with IBD and without IBD using OC-FCa and compared it with Phadia 250 (Thermo Fisher Scientific, Freiburg, Germany), which is a fluorescence immunoassay widely used to test for f-Cal [12,13,14,15]. Moreover, we evaluated the agreement and diagnostic performance of OC-FCa and Phadia 250. To the best of our knowledge, there is no study of the comparison of f-Cal between OC-FCa and Phadia 250.

## 2. Materials and Methods

### 2.1. Study Population

A total of 278 stool samples were collected, of which 158 were obtained from patients with suspected IBD from 1 August 2022 to 30 June 2023 at Konkuk University Medical Center, Seoul, Korea. The study population for IBD evaluation comprised 81 men (51.3%) and 77 women (48.7%), with 55 adults (median age, 39; interquartile range (IQR), 29.5–54.5) and 103 children (median age, 8; IQR, 4–13). As there were no significant differences in f-Cal concentrations between adults and children depending on the clinical outcome, the study population was not intentionally classified into age groups. According to medical records, the clinical outcomes of the 158 samples were classified into 43 IBD (7 Crohn’s disease, 35 ulcerative colitis, and 1 eosinophilic colitis) and 115 non-IBD samples based on the results of radiology, endoscopy, and histology. The medical records of each patient for diagnosis and/or medical conditions were reviewed retrospectively. Patients with IBD who achieved remission were excluded for study accuracy. An additional 120 samples were obtained from an apparently healthy FIT-negative adult population who underwent a general medical examination (65 men and 55 women, median age, 51; IQR, 41–58). This study was approved by the Institutional Review Board of Konkuk University Medical Center, Seoul, Korea (KUMC 2023-03-057, 8 August 2023) The patient identifiers were sys-tematically omitted and there was no study-specific intervention because residual samples were used after the requested stool analysis. Therefore, obtaining written informed consent was waived. The residual samples for our study were stored in a −70 °C refrigerator for one–three months. Before analysis, frozen samples were thawed at room temperature (20 to 25 °C).

### 2.2. f-Cal Assay

All study samples were analyzed using OC-FCa and Phadia 250. The measurement principle of OC-FCa is latex turbidimetry using a latex agglutination reaction [9,11]. Latex agglutination is an antigen–antibody reaction involving antigen- or antibody-sensitized polystyrene latex particle clumping [9]. When the latex reagent (OC-FCa R-2 Latex Reagent) for OC-FCa coated with anti-human calprotectin antibodies is mixed with calprotectin in an IBD sample, the aggregates are formed [16]. When a light beam passes through the reaction liquid, the aggregates decrease the intensity of the transmitted light beam [9]. These changes present a response curve of absorbance units vs. calprotectin concentration, and the concentration of calprotectin in the IBD sample is determined from this curve [9,16]. Before measuring calprotectin in stool samples, OC-FCa creates a calibration curve of six points using the OC-FCa calibrator and checks the control values using the OC-FCa control [17]. OC-FCa analyzes stool samples using an OC-auto sampling bottle 3, which is diluted by mixing 10 mg of collected stool samples with 2 mL of buffer [16]. Stool samples were analyzed using OC-FCa, according to the manufacturer’s instructions. The response curve obtained from the antigen–antibody reaction was compared to the calibration curve. The f-Cal concentration was interpreted as positive or negative according to the cut-off value specified by the manufacturer (>50 μg/g, positive; ≤50 μg/g, negative) [16].

Phadia 250 uses EliA Calprotectin 2, a fluoroenzyme immunoassay, for calprotectin measurement [12]. Monoclonal antibodies coated with EliA Calprotectin 2 bind to the f-Cal in stool samples. The EliA Calprotectin 2 conjugate, an enzyme-labeled antibody against calprotectin, is added to form a calprotectin–conjugate complex [12]. After the calprotectin–conjugate complex is incubated with a development solution, the level of fluorescence in the reaction mixture is measured. After confirming the control values using both a positive and negative control, along with curve control strips, the Phadia 250 creates a calibration curve [12]. Stool samples were also analyzed using a Phadia 250 according to the manufacturer’s instructions. Results were interpreted as positive or negative according to the cut-off value specified by the manufacturer (>50 μg/g, positive; ≤50 μg/g, negative) [12].

F-Cal concentrations over the analytical measurement range (AMR) were recorded as the lower and upper limits of the AMR for each analyzer. The AMRs of OC-FCa and Phadia 250 were 20–2720 and 11.5–2000, respectively.

### 2.3. Statistical Analysis

The distribution of f-Cal concentration was tested for normality using the Kolmogorov–Smirnov Z-test. Reference intervals were defined using a non-parametric method, according to the clinical and laboratory standards institute (CLSI), guideline EP28-A3c [18]. The outliers were checked using Reed’s criterion [19]. Lower and upper reference limits and 90% confidence intervals (CI) were estimated using a non-parametric method. The agreement between OC-Sensor Pledia and Phadia 250 was calculated using Cohen’s kappa (κ) with 95% CI, which was interpreted as follows: ≤0.20, none; 0.21–0.39, minimal; 0.40–0.59, weak; 0.60–0.79, moderate; 0.80–0.90, strong; and >0.90, nearly perfect [20]. A Bland–Altman plot and Passing–Bablok regression analysis were performed to compare OC-FCa and Phadia 250, according to CLSI guideline EP09C-ED3 [21]. The absolute mean difference in f-Cal between OC-FCa and Phadia 250 was analyzed using a Bland–Altman plot. F-Cal levels between OC-FCa and Phadia 250 were compared using Passing–Bablok regression. Pearson’s correlation coefficients (r) with 95% CIs were interpreted as follows: <0.30, negligible; 0.30–0.50, low; 0.50–0.70, moderate; 0.70–0.90, high; 0.90–1.00, very high [22]. A receiver operating characteristic (ROC) curve was used to estimate the optimal cut-off for differentiating patients with IBD from those without IBD. The areas under the ROC curve (AUCs) with 95% CIs were calculated and distinguished as follows: AUC = 0.5, non-informative; AUC = 0.5–0.7, less accurate; AUC = 0.7–0.9, moderately accurate; AUC = 0.9–1.0, highly accurate; and AUC = 1.0, perfect [23]. Statistical analyses were performed using Microsoft Excel Software (version 2016; Microsoft Corporation, Redmond, WA, USA) and MedCalc Statistical Software (version 22.013; MedCalc Software, Ostend, Belgium); *p* < 0.05 was considered statistically significant.

## 3. Results

### 3.1. Distribution of f-Cal Levels in Each Population: OC-FCa and Phadia 250

**Figure 1:**
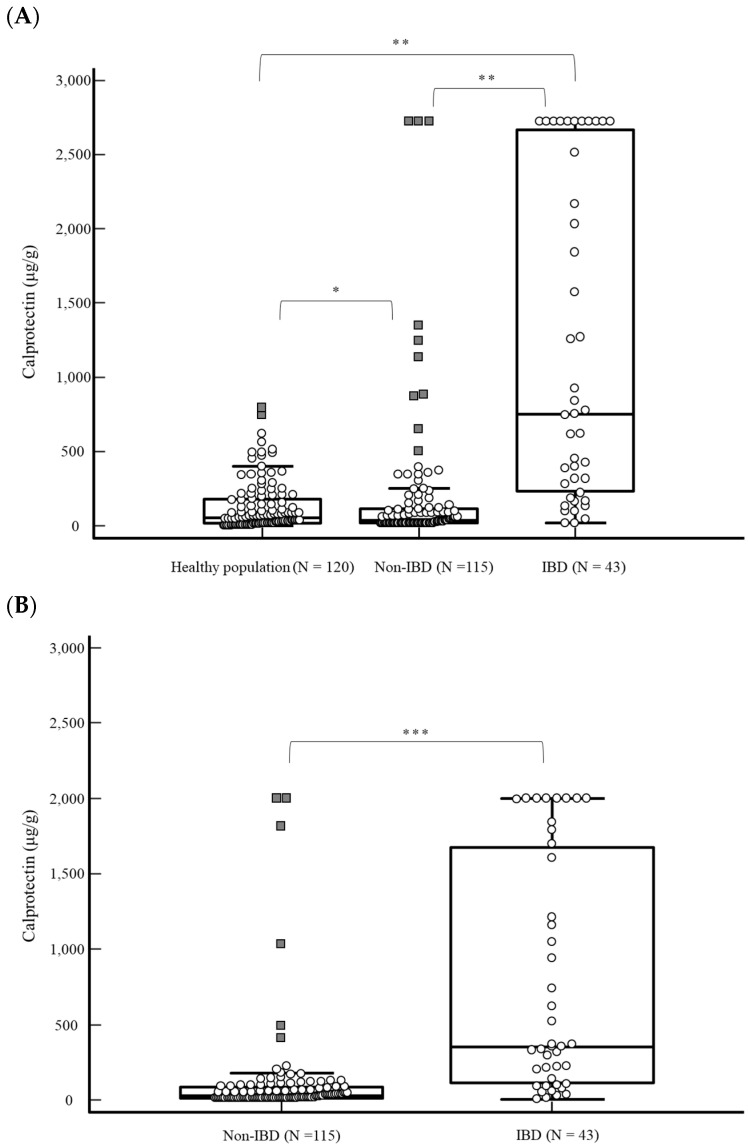
(**A**) Fecal calprotectin concentration measured using OC-Sensor Pledia in healthy population and patients without and with IBD (median = 56.5, 34, and 751 μg/g, respectively). (**B**) Fecal calprotectin concentration measured using Phadia 250 in patients without and with IBD (median = 29.0 and 355.0 μg/g, respectively). * *p* = 0.1394, ** *p* < 0.0001, *** *p* < 0.001. Abbreviations: IBD, inflammatory bowel disease; N, number.

All 278 fecal samples were used for distribution analysis without any outliers. The distribution of f-Cal using OC-FCa in healthy populations and patients with IBD and without IBD is shown in Figure 1A. The measurement of f-Cal in healthy populations was performed using only OC-FCa. The median f-Cal concentrations (IQR) using OC-FCa were 56.5 μg/g (17–175.5) in the healthy population, 34 μg/g (20–112.5) in patients without IBD, and 751 μg/g (250.5–2616.5) in patients with IBD. The f-Cal concentration in patients with IBD was significantly different from that in patients without IBD and the healthy population (*p* < 0.0001). The distribution of f-Cal measured using Phadia 250 in patients with and without IBD is shown in Figure 1B. The median f-Cal concentrations (IQR) using Phadia 250 were 29.0 μg/g (15.5–42.5) and 355.0 μg/g (225.3–983.6) in patients without and with IBD, respectively. F-Cal concentrations were significantly different between patients with and without IBD (*p* < 0.001).

**Figure 2:**
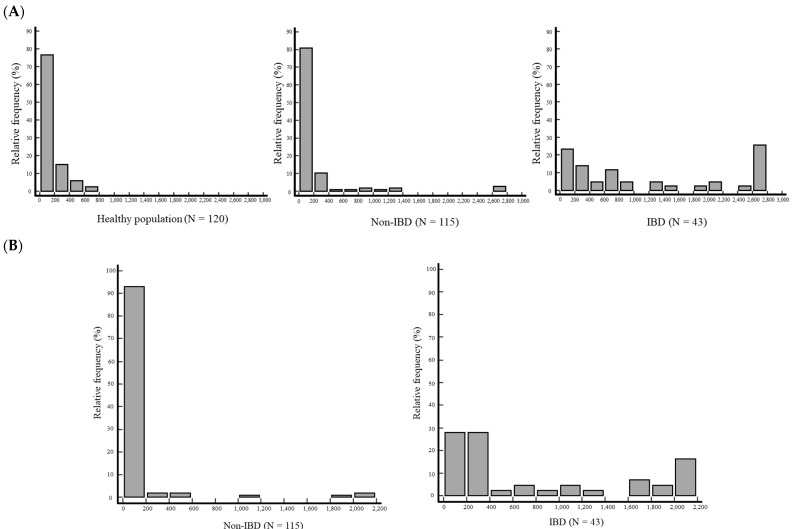
(**A**) Histograms of fecal calprotectin measured using OC-Sensor Pledia in the healthy population and patients without and with IBD. (**B**) Histograms of fecal calprotectin measured using Phadia 250 in patients without and with IBD. Abbreviations: IBD, inflammatory bowel disease; N, number.

The distributions of f-Cal in the healthy population and patients without IBD measured using OC-FCa were non-Gaussian and showed positively skewed histograms (Figure 2A). In both OC-FCa and Phadia 250, f-Cal concentrations in patients with IBD showed a bimodal distribution (Figure 2A,B).

According to the manufacturer cut-off value (>50 μg/g), 51.7% of f-Cal levels in the healthy population exceeded the cut-off value. Furthermore, 43.5% and 37.4% of f-Cal levels measured using OC-FCa and Phadia 250, respectively, in patients without IBD exceeded the cut-off value.

### 3.2. Qualitative and Quantitative Comparison of Two f-Cal Assays

Agreement between OC-FCa and Phadia 250 for f-Cal was determined according to the manufacturer cut-off value (>50 µg/g). The concordance between OC-FCa and Phadia 250 was 82.3% with moderate agreement (kappa = 0.644, 95% CI = 0.525–0.763) (Table 1).

**Figure 3:**
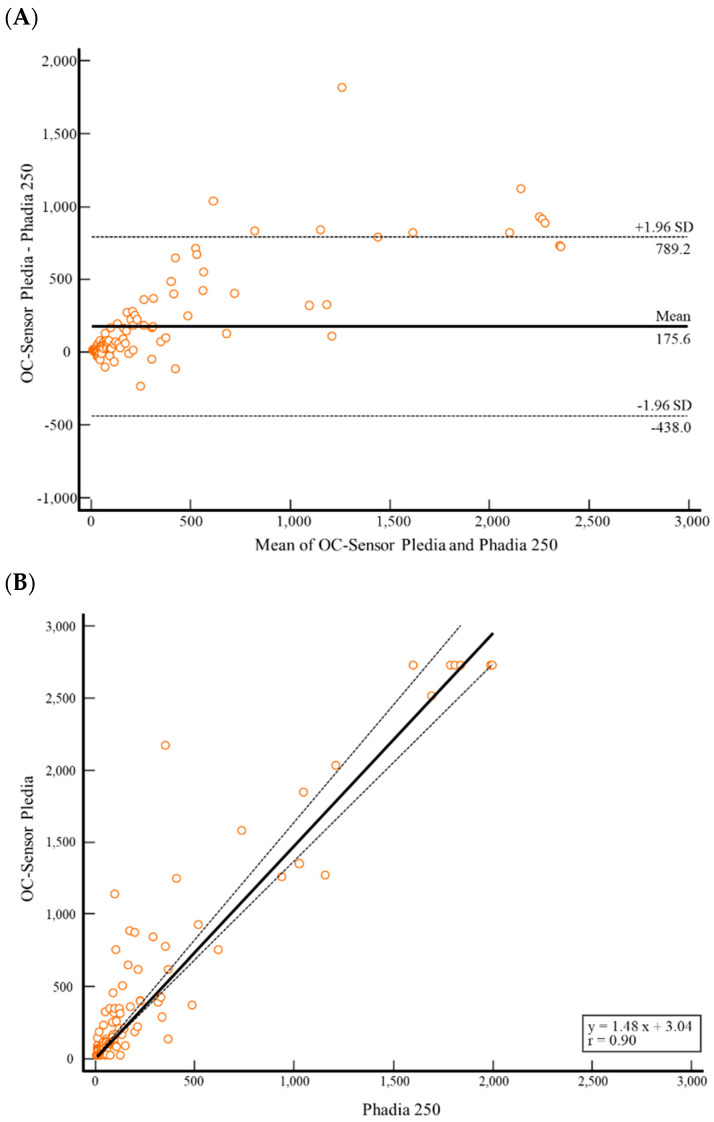
Comparison of fecal calprotectin measurement between OC-Sensor Pledia and PHADIA 250 using Bland–Altman Plot (**A**) and Passing–Bablok regression (**B**) (N = 158). Solid line, mean difference or Passing–Bablok regression; dashed line, ±1.96 SD or 95% CI. Abbreviations: CI, confidence interval; SD, standard deviation; N, number.

The absolute mean difference of f-Cal was 175.6 (95% CI, 126.4–224.8) and ranged from −438.0 to 789.2 between OC-FCa and Phadia 250 (Figure 3A). The mean difference (%) of f-Cal was 46.0% (95% CI, 37.6–54.4) and ranged from −58.5% to 150.5% between OC-FCa and Phadia 250. In the Passing–Bablok correlation, the f-Cal values of OC-FCa and Phadia 250 showed a high correlation (r = 0.90) (Figure 3B).

### 3.3. Diagnostic Performance of Two f-Cal Assays

**Figure 4:**
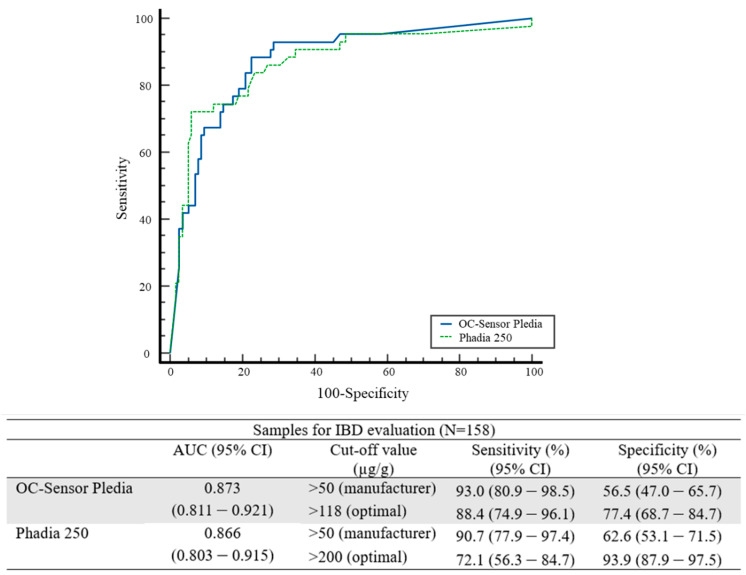
Comparison of clinical performances of OC-Sensor Pledia and Phadia 250 using the receiver operating characteristics for diagnosing inflammatory bowel disease. Abbreviations: IBD, inflammatory bowel disease; N, number; AUC, areas under the ROC curve; CI, confidence interval.

OC-FCa and Phadia 250 showed moderate accuracy (AUC = 0.873 and 0.866, respectively) in diagnosing IBD; however, they did not show a significant difference (*p* = 0.616) (Figure 4). Based on the manufacturer’s cut-off value, the sensitivities of OC-FCa and Phadia 250 were 93.0% and 90.7%, respectively, and the specificities of OC-FCa and Phadia 250 were 56.5% and 62.6%, respectively. According to the optimal cut-off values, the sensitivities of OC-FCa and Phadia 250 decreased (88.4% and 72.1%, respectively); however, their specificities improved (77.4% and 93.9%, respectively) (Figure 4).

## 4. Discussion

F-Cal is a useful biomarker for the differentiation between patients with IBD and without IBD, and the evaluation of IBD courses [2,4,5]. F-Cal is also more convenient than endoscopy for initial IBD screening [2,5]. Various methods exist for f-Cal detection, including chemiluminescent, enzyme-linked immunosorbent, fluorescent, and immuno-turbidimetric assays [2]. We analyzed f-Cal levels using a latex agglutination turbidimetric immunoassay, OC-FCa, in healthy individuals and patients with IBD and without IBD. Moreover, we compared OC-FCa and Phadia 250. Currently, immunoassays such as Phadia 250 are the most preferred and widely utilized methods for fecal calprotectin testing. The OC-Sensor Pledia, while similarly priced, offers notable advantages. As previously mentioned, it allows patients to collect samples directly into the container, which can enhance sample stability. Furthermore, it facilitates testing without the need for additional aliquoting in the laboratory, thereby streamlining the testing process.

In the OC-FCa, the f-Cal of patients with IBD was significantly higher than that of the healthy population and patients without IBD (*p* < 0.0001) (Figure 1A). There was no significant difference in f-Cal levels between the healthy population and patients without IBD (*p* = 0.1394). F-Cal levels did not increase in patients without IBD compared to the healthy population, even though they had gastrointestinal symptoms. These findings are consistent with the results of many studies, namely that f-Cal supports the differential diagnosis of IBD and functional bowel disorders [2,3,4,5,6]. However, over half of the f-Cal levels in the healthy population and over one-third of the f-Cal levels in patients without IBD exceeded the manufacturer’s cut-off value. The manufacturer’s cut-off value might low to increase sensitivity for screening purposes, and it is important for clinicians to be aware of this aspect.

The qualitative and quantitative comparisons of OC-FCa and Phadia 250 showed acceptable results (Table 1 and Figure 3). Although the agreement was moderate (kappa = 0.644), the results can be different depending on the ratio of positive to negative. Quantitatively, f-Cal detected using OC-FCa was higher than that detected using Phadia 250. However, the two assays showed a high correlation (r = 0.90) (Figure 3B). Laboratories have reported f-Cal not only as qualitative but also as quantitative results. Because the cut-off value of f-Cal is not an absolute diagnostic criterion for IBD, endoscopy and clinical correlation are required for IBD diagnosis. Therefore, physicians must consider the trends in each assay for f-Cal.

The AUCs between OC-FCa and Phadia 250 were not significantly different (*p* = 0.616) (Figure 4). For both the manufacturer and optimal cut-off values, the sensitivity of OC-FCa was higher than Phadia 250, and the specificity of OC-FCa was lower than that of Phadia 250. There is no reference f-Cal cut-off value for differentiating patients with and without IBD [5]. Therefore, many studies have shown different optimal cut-off values depending on the device used [6,11,13,24,25,26]. However, various manufacturers suggest a cut-off value > 50 µg/g for f-Cal [12,13,16]. Possibly, the manufacturer’s cut-off value is much lower than the optimal cut-off values for IBD screening. According to the manufacturer’s cut-off, further evaluation is needed for the diagnosis of IBD in over half of the healthy population. Because of the low specificity of the manufacturer’s cut-off, f-Cal should be used along with endoscopic results for IBD diagnosis. In 2021, as part of the process for selecting therapeutic targets in inflammatory bowel disease, the international organization for the study of inflammatory bowel diseases recommended a gray zone of f-Cal, ranging from 150 to 250 μg/g because of the low reliability of f-Cal [27].

Two previous studies have evaluated the performance and optimal cut-off value of OC-FCa [10,11]. In our study, the optimal cut-off value (>118 µg/g) of f-Cal was much lower than a previous study (>600 µg/g) [11]. This result could be explained by the difference in the proportion of patients with IBD in the study population and the severity of IBD.

Several studies have compared Phadia 250 with other devices for f-Cal detection [13,14,15,25,26]. These studies demonstrated that Phadia 250 had good analytical and diagnostic performance, and showed various optimal cut-off values depending on the studies using Phadia 250 and other devices [13,14,25,26]. These findings might also be attributed to the lack of a standardized cut-off value for f-Cal; therefore, cut-off values can vary depending on the study population and the method used in laboratories.

This study had several limitations. First, evaluating f-Cal concentration depending on the severity of IBD was difficult because of the small number of patients with IBD. To determine the correlation between IBD severity and f-Cal concentration, a larger sample size of patients with IBD is required. Second, we underestimated the factors that could affect f-Cal, including other gastrointestinal inflammation [4], temperature during sample storage [28,29,30], and the buffer used for the test [24]. Although f-Cal is a useful method for reducing unnecessary procedures in clinical practice, the interpretation of f-Cal should consider various factors that can affect its concentration.

In conclusion, this is the first study to comprehensively compare f-Cal levels between OC-FCa and Phadia 250. We also compared the f-Cal distribution in healthy individuals and patients with and without IBD. Both OC-FCa and Phadia 250 showed high correlations and good clinical performance, and f-Cal measured by OC-FCa was reliable for the initial differentiation between patients with and without IBD. Therefore, OC-FCa and Phadia 250 can be alternative methods for measuring f-Cal, depending on the laboratory situation.

## Figures and Tables

**Table 1:** Agreement of fecal calprotectin concentration between OC-Sensor Pledia and Phadia 250.

Fecal Calprotectin Concentration		OC-Sensor Pledia		Total	Kappa (95% CI)	Concordance (95% CI)
		≤50 µg/g	>50 µg/g			
Phadia 250	≤50 µg/g	58	18	76 (48.1%)	0.644 (0.525–0.763)	82.3% (75.4–87.9)
	>50 µg/g	10	72	82 (51.9%)		
Total		68 (43.0%)	90 (57.0%)	158		

Abbreviations: CI, confidence interval.

## Data Availability

The original contributions presented in the study are included in the article, further inquiries can be directed to the corresponding author.
